# Clinical Utility of Plasma *p*‐tau217 Compared to PET and CSF in the Diagnosis of Cognitive Disorder

**DOI:** 10.1002/alz70856_102622

**Published:** 2025-12-25

**Authors:** Yu‐Yuan Huang, Yu Guo, Yi‐Han Gan, Yi‐Lin Chen, Rui‐Xin Yao, Ling‐Ling Li, Jin‐tai Yu

**Affiliations:** ^1^ Department of Neurology and National Center for Neurological Disorders, Huashan Hospital, State Key Laboratory of Medical Neurobiology and MOE Frontiers Center, Fudan University, Shanghai, Shanghai, China; ^2^ Huashan Hospital, Fudan University, Shanghai, Shanghai, China; ^3^ Department of Neurology and National Center for Neurological Disorders, Huashan Hospital, State Key Laboratory of Medical Neurobiology and MOE Frontiers Center, Fudan University, Shanghai, China; ^4^ National Center for Neurological Disorders, Shanghai, China; ^5^ Huashan hospital, Fudan University, Shanghai, China

## Abstract

**Background:**

The real‐world utility and impact of plasma *p*‐tau217 on diagnosing various cognitive disorders remains unknown.

**Method:**

To evaluate whether adding plasma *p*‐tau217 to the standard clinical workup leads to diagnostic revisions and increased diagnostic confidence, we included 622 unselected individuals who sought evaluation at the memory clinic and were diagnosed using positron emission tomography (PET) or cerebrospinal fluid (CSF) examinations.

**Result:**

Incorporating plasma *p*‐tau217 in the diagnostic workup significantly impacted clinical decisions, leading to diagnostic revisions for 30.06% of participants and enhancement in overall diagnostic confidence from 64.70% to 78.92%. Compared to final diagnoses, plasma *p*‐tau217 demonstrated a sensitivity of 0.88 and a specificity of 0.98 for Alzheimer's disease (AD), outperforming routine diagnostic procedures. Plasma *p*‐tau217 performance was comparable to PET or CSF tests across various cognitive stages, including subjective cognitive decline, mild cognitive impairment, and dementia. For non‐AD diagnosis, plasma *p*‐tau217 markedly influenced the clinical diagnosis of frontotemporal dementia (*P* < 0.001), vascular dementia (*P* < 0.001), α‐synucleinopathies (*P* < 0.001), and potential non‐AD tauopathies (*P* = 0.001), showing diagnostic performance similar to PET imaging.

**Conclusion:**

In conclusion, plasma *p*‐tau217 significantly impacts the diagnosis and diagnostic confidence of cognitive impairment, offering a less invasive, cost‐effective, and accessible alternative to CSF and PET examinations. Its integration into clinical practice holds promise for streamlining diagnostic procedures and optimizing medical resources in real‐world memory clinics.

